# Metagenomics reveals effects of fluctuating water conditions on functional pathways in plant litter microbial community

**DOI:** 10.1038/s41598-023-49044-x

**Published:** 2023-12-08

**Authors:** Matevž Likar, Mateja Grašič, Blaž Stres, Marjana Regvar, Alenka Gaberščik

**Affiliations:** 1 Biotechnical Faculty, Department of Biology, University of Ljubljana, Večna Pot 111, 1000 Ljubljana, Slovenia; 2 Institute of Sanitary Engineering, Faculty of Civil and Geodetic Engineering, University of Ljubljana, Ljubljana, Slovenia; 3 Biocybernetics and Robotics, Department of Automation, Jožef Stefan Institute, Ljubljana, Slovenia; 4 Department of Catalysis and Chemical Reaction Engineering, National Institute of Chemistry, Ljubljana, Slovenia

**Keywords:** Bacteria, Microbial communities, Environmental microbiology, Fungi, Climate-change impacts, Wetlands ecology

## Abstract

Climate change modifies environmental conditions, resulting in altered precipitation patterns, moisture availability and nutrient distribution for microbial communities. Changes in water availability are projected to affect a range of ecological processes, including the decomposition of plant litter and carbon cycling. However, a detailed understanding of microbial stress response to drought/flooding is missing. In this study, an intermittent lake is taken up as a model for changes in water availability and how they affect the functional pathways in microbial communities of the decomposing *Phragmites australis* litter. The results show that most enriched functions in both habitats belonged to the classes of Carbohydrates and Clustering-based subsystems (terms with unknown function) from SEED subsystems classification. We confirmed that changes in water availability resulted in altered functional makeup of microbial communities. Our results indicate that microbial communities under more frequent water stress (due to fluctuating conditions) could sustain an additional metabolic cost due to the production or uptake of compatible solutes to maintain cellular osmotic balance. Nevertheless, although prolonged submergence seemed to have a negative impact on several functional traits in the fungal community, the decomposition rate was not affected.

## Introduction

Wetlands play an essential role in the global carbon cycle, which depends on their primary productivity and decomposition rate^1^. The accumulation and decomposition of plant litter from vegetation in wetlands are very variable, as plant material and environmental conditions may be highly heterogeneous^2^. This variability of environmental conditions will become even more pronounced due to climate change, leading to increased precipitation and atmospheric moisture^3^ due to changes in atmospheric circulation, a more active hydrological cycle, and increased water-holding capacity of the atmosphere^4^. The hydrological cycle intensification will likely increase the intensity of extreme precipitation events and the risk of flooding^5^. However, local low precipitation extremes can also result from climate change^3^. Such changes in moisture availability are believed to affect a range of ecological processes, including the decomposition of plant litter and carbon cycling. Wetlands present an important carbon sink; however, climate change and human interventions can turn them into carbon sources^6^.

Lake Cerknica (Slovenia) is an intermittent wetland with pronounced water level fluctuations^2^. Water level fluctuations severely affect decomposition and microbial colonization in such intermittent ecosystems^7,8^. Periodic flooding may cause hypoxic or anoxic conditions and thus impact microbial activity and the decomposition of plant litter^2^. On the other hand, water and carbon may be limiting factors for bacteria and fungi in wetlands with low soil water content^6^; thus, severe drying during the low precipitation phase also has adverse effects on the decomposition rates^9^. Therefore, Lake Cerknica presents a unique system to obtain insight into the impact of different water regimes on microbial activity and their involvement in carbon cycling.

Wetland communities, including common reed (*Phragmites australis*), characterize the vegetation of Lake Cerknica^10^. *P. australis* contributes significantly to the primary production of the area and, consequently, to the production of litter in the ecosystem^7^. Its high biomass production makes it a good candidate for decomposition studies of plant litter in wetland conditions. Microbial colonization and decomposition of *P. australis* leaves starts already during the vegetation season^11–13^, with the phyllosphere community forming a significant portion of decomposers in the early decomposition phase^13^. Decomposition theories describe fungi as primary decomposers of plant litter^14^, as they break down the lignocellulose matrix^15^, followed by bacteria that are regarded as decomposers of detrital particulate and dissolved organic matter^16^. In contrast, recent observations revealed that both microbial groups are essential players in all stages of the decomposition process^17^. However, the precise contributions of fungi and bacteria to the decomposition process remain uncertain, and this can depend on a variety of factors, including the quality of carbon (C) inputs from plants (e.g.^18,19^). Fungi often prefer to utilize more resistant and complex C sources, while bacteria respond rapidly to increased resource availability and colonize readily available, labile C resources^20,21^. On the other hand, when more easily degradable substrates are available, the dominance within the microbial community can shift from fungi to bacteria^22^. Nevertheless, the relationship between substrate C quality and the microbial community is not straightforward, and both fungi and bacteria can employ mixed strategies to exploit available substrates^23,24^. Furthermore, both microbial groups seem to respond differently to changes in water availability, with bacteria typically responding faster than fungi to changes in soil water availability^25^. Yang et al.^26^ observed that the bacterial community was more sensitive to changes in precipitation than the fungal community, which was more sensitive to inter-annual differences in precipitation but not to the treatment-induced changes in precipitation. Shifting precipitation regimes in the future climate could thus alter the stability and functions of microbial communities. With this in mind, parallel analysis of both microbial groups is vital to fully understand the dynamics of microbial communities in the decomposing litter.

Our study aimed to evaluate the effect of changed environmental conditions (dry and wet) due to fluctuating water levels on the functional traits of microbial communities of *P. australis* litter. To predict litter decomposition dynamics in changing environments, we must understand changes in microbial functional pathways under fluctuating water conditions. With this aim, we analysed the functional traits of microbial communities in fresh leaves and leaves after a 45-day decomposition phase in either wet or dry habitats characterized by different flooding patterns. First, we hypothesized that functional traits in the microbial communities from fresh leaves would change due to the advancement of the decomposition processes. Second, we hypothesized that the response of communities developed on leaf litter deposited in different habitats would differ relative to their submergence level, showing differences in the metabolic processes. Our last hypothesis was that differences in the water regimes between both habitats would affect the functional traits of bacteria and fungi in the decomposing litter differently.

## Materials and methods

### Site description

Lake Cerknica is one of the largest intermittent lakes in Europe (29 km^2^) and a Ramsar and Natura 2000 (https://natura2000.gov.si/en/) site. The climate in the area is continental, with a mean temperature of 9.2 °C and annual precipitation of about 1700 mm. On average, the site is flooded nine months per year. The extent and duration of these floodwaters result in various habitats that change over time. *Phragmites australis* thrives in different parts of the inner lake and the edge of the lake along the lake tributaries^2^.

### Plant material collection and decomposition experiment

For the present study, we collected lower leaves (the bottom five leaves on the plant) and upper leaves (the topmost five fully developed leaves) of *P. australis* at the end of the vegetation period, when plants were still green, in the lake area at Zadnji Kraj (45°44′27.60" N, 14°22′12.62" E)^13^. Plants were sampled in compliance with relevant institutional, national, and international guidelines and legislation. All necessary permissions and licences were obtained before the collection. A voucher specimen was deposited in the Herbarium of the University in Ljubljana under identification LJU10147538. The material was collected and identified formally by the corresponding author (Matevž Likar). Four g of collected leaves were filled into litter bags (17 cm × 22 cm, made from 1 mm × 1 mm plastic mesh) and exposed at a predominantly dry (45°43′38.29" N, 14°24′16,72" E) and a mainly wet (45°43′38.30" N, 14°24′16,59" E) site in the vicinity of Gorenje Jezero for 45 days, as described in^13^. For each treatment, five parallel samples were exposed to decomposition. After 45 days, samples from each bag were homogenized by mixing and 0.2 g aliquots were collected and frozen at -80 °C until further processing. After that allochthonous material (e.g. small invertebrates and sand grains) were removed and the remaining plant material was oven dried to the constant weight. The decomposition rate was calculated as a percentage of the dry weigh of the initial plant material. The plant litter decomposition percentage was, on average, 32% for the samples in the wet habitat and 14% for the samples in the dry habitat.

### Sample processing and bioinformatics pipeline

The DNA was extracted from fresh common reed leaves and leaves after the initial 45-day decomposition using the GenElute® Plant Genomic DNA isolation kit (Sigma), following the manufacturer's instructions. DNA was sequenced at Macrogen, Inc. (South Korea) using an Illumina HiSeqX platform. Paired-end libraries were prepared using the TruSeq Nano kit (Illumina) kit. The read length was 150 bp. Analysis and annotation of output data were performed through Metagenomics rapid annotation (MG-RAST) online server version 4.0.3^27^ with the default parameters using BLAT algorithm^28^ against M5nr and M5rna databases^29^. Functional classification was performed using the SEED Subsystems database. Taxonomic annotations up to genus level for fungi and family level for bacteria were performed using the RefSeq database with a maximum e-value cut-off of 10^−5^, minimum identity cut-off of 60% and a minimum sequence alignment length of 15 nucleotides. Data matrices derived from MG- RAST were imported into R for downstream analyses.

### Statistical analysis

All analyses were performed in R v4.2.0. Rarefaction, ordination and differential abundance analyses of the datasets were performed using the phyloseq (v1.36.0) and DESeq2 (v1.32.0) libraries. Results were visualised using ggplot2 (v3.3.5) and complexHeatmap (v2.8.0) libraries.

Functional beta diversity of the communities was explored by analysing differences in abundances of SEED Subsystems annotations among habitats and leaf position. It was visualised by a redundancy analysis (RDA) based on Bray–Curtis distance. The differences between the grouping variables were evaluated with permutational analysis of variance (perMANOVA, using the *adonis2* function in the vegan v2.5–7 library) based on 999 permutations and Bray–Curtis distance. General perMANOVA was followed by the *pairwise.adonis* function (https://github.com/pmartinezarbizu/pairwiseAdonis.git). Permutational analysis of multivariate dispersion using the *betadisper* function with 9999 permutations was used to evaluate the perMANOVA results.

SIMPER analysis in the R vegan library was used to determine the similarity or difference between the abundance distributions of SEED Subsystems among representative metagenomic datasets from fresh leaves and leaves decomposing in either dry or wet habitats. This enabled us to calculate the partition of the average Bray–Curtis dissimilarity between groups of samples into components from different Subsystems categories and allowed the identification of categories that were most important in creating the observed patterns in dissimilarity. SIMPER analysis was performed using 9,999 permutations.

## Results

The functional analysis yielded, on average, 3,080,580 (459,115–4,542,089; min–max) and 28,204 (3,129–77,254; min–max) sequences annotated with function according to the SEED Subsystems classification for bacterial and fungal communities, respectively. This represents 64% of all identified bacterial sequences and 4% of all fungal sequences in our dataset.

**Figure 1:**
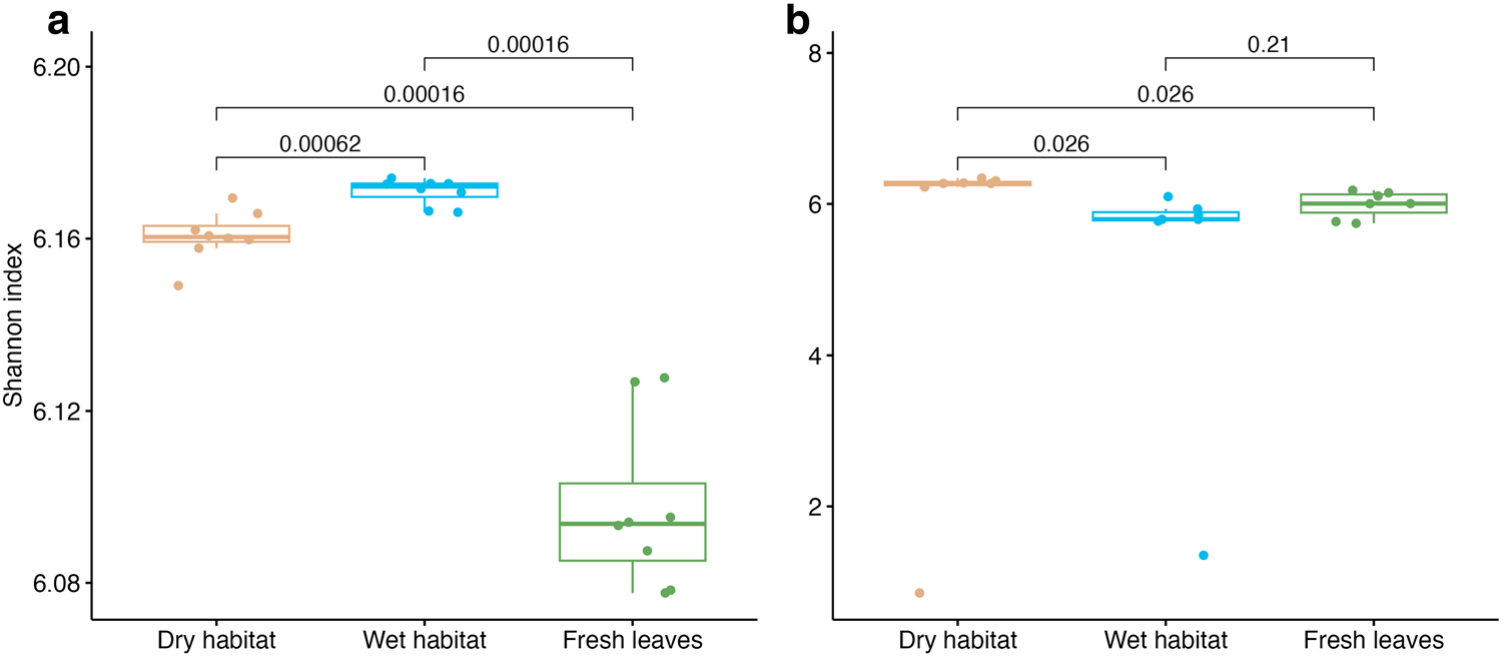
Shannon index for functional genes annotated with Subsystems at the level of function for (**a**) bacterial and (**b**) fungal communities. The numbers above boxplots represent p-values of pairwise Wilcox tests.

Bacterial and fungal communities demonstrated similar functional α–diversity (Fig. 1), with some differences between different habitats/fresh leaves. For bacterial communities, functional α–diversity between fresh and decomposing leaves, as well as between leaves decomposing in different habitats, differed significantly, with the lowest diversity observed in fresh leaves. In contrast, no difference in functional α–diversity was observed between fresh leaves and leaves decomposing in the wet habitat for fungal communities. Dry habitat fungal communities in decomposing leaves showed the highest functional diversity, which was also statistically significant from wet and fresh habitats.

### Bacterial communities

**Figure 2:**
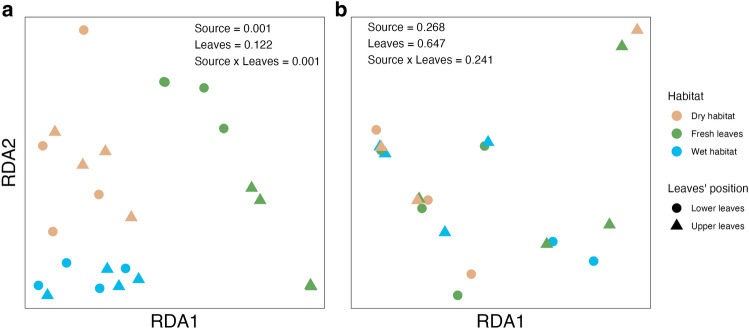
Redundancy analysis of (**a**) bacterial and (**b**) fungal functional communities on fresh leaves or leaves decomposing in dry/wet habitats. The values display the overall perMANOVA p-values for the habitat, leaves' position or both as the categorical variable.

Redundancy analysis at the level of function in SEED Subsystems annotations showed good separation in bacterial physiology between fresh leaves and leaves decomposing in either dry or wet habitats (Fig. 2a). perMANOVA confirmed the differences between fresh and decomposing leaves obtained by redundancy analysis (*p* = 0.001), whereas there was no overall statistically significant difference between functional traits of bacterial communities on litter decomposing in wet or dry habitats (*p* = 0.060). The initial position of leaves on the plant did not seem to affect the bacterial communities (*p* = 0.122). Nevertheless, the interaction between both variables was found; therefore, we tested the effect of leaf position individually for fresh leaves and leaves decomposing in an individual habitat. Only fresh leaves showed a significant difference at *p* = 0.001 between leaves of different leaf positions.

**Figure 3:**
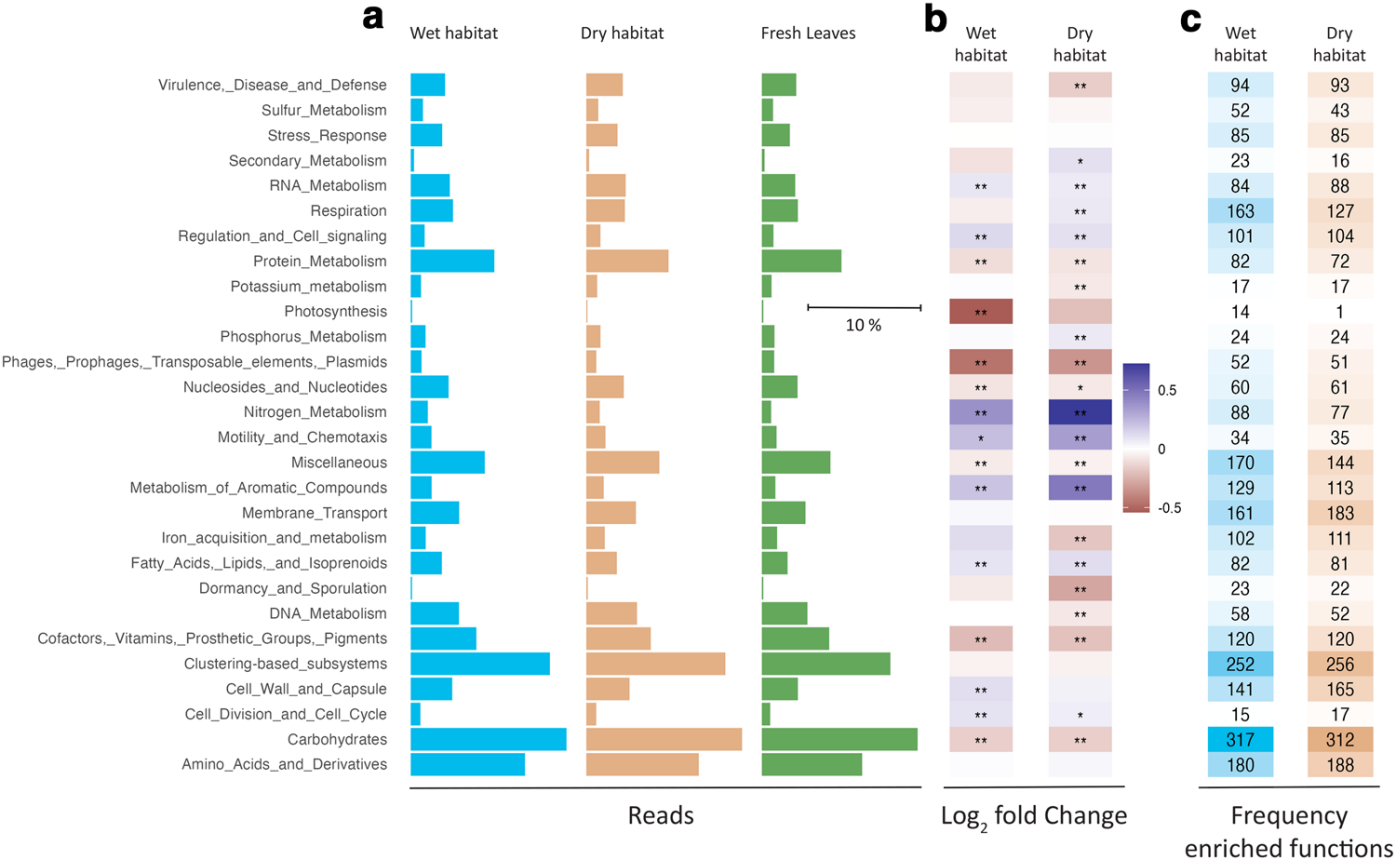
Effect of decomposition on bacterial physiology at level 1 of the SEED Subsystems classification: relative abundance of reads for fresh leaves or leaves decomposing in wet/dry habitat; DESeq analysis of reads from each habitat compared to fresh leaves; frequencies of enriched functions (*p* < 0.05) in wet/dry habitat.

In total, 8021 SEED Subsystems functions were annotated. The highest number of reads was observed for Protein metabolism, Clustering-based subsystems (functional coupling evidence among the proteins, but their exact roles in the metabolic pathways are yet unknown), Carbohydrates and Amino acids and derivates (Fig. 3a). This was true for fresh leaves and for leaves decomposing in wet or dry habitats, as all three exhibited similar sequencing profiles for level 1 annotations. Despite similar overall patterns between treatments, the abundances in the individual functional classes showed either increased or decreased abundance in decomposing leaves when compared to fresh leaves (Fig. 3b). The highest decrease in the wet habitat was observed for Photosynthesis and the highest increase for Nitrogen metabolism. Nitrogen metabolism showed an even higher increase in the dry habitat. In contrast, the most significant decreases were observed for Phages, Prophages, Transposable elements and Plasmids, and the Dormancy and Sporulation categories. Several functional traits at level 1 showed significant differences between leaves decomposing in dry vs wet habitat (Suppl. Mat. Table S1).

Most functions enriched in decomposing leaves in both habitats belonged to classes of Carbohydrates and Clustering-based subsystems (Fig. 3c). Other Subsystems categories showed similar enrichment patterns for both habitats. Still, they were generally lower in the dry habitat. Spearman coefficients for enriched and under-enriched functions in wet and dry habitats were above 0.98. SIMPER analysis revealed that functions in Carbohydrate metabolism contributed 24% and 39% of the dissimilarity between fresh leaves and leaves decomposing in dry or wet habitats, respectively. The dissimilarity between leaves decomposing in wet vs dry habitats was due to Carbohydrates (24%), Nitrogen metabolism (16%), Respiration (16%), and Metabolism of aromatic compounds (16%).

### Fungal communities

**Figure 4:**
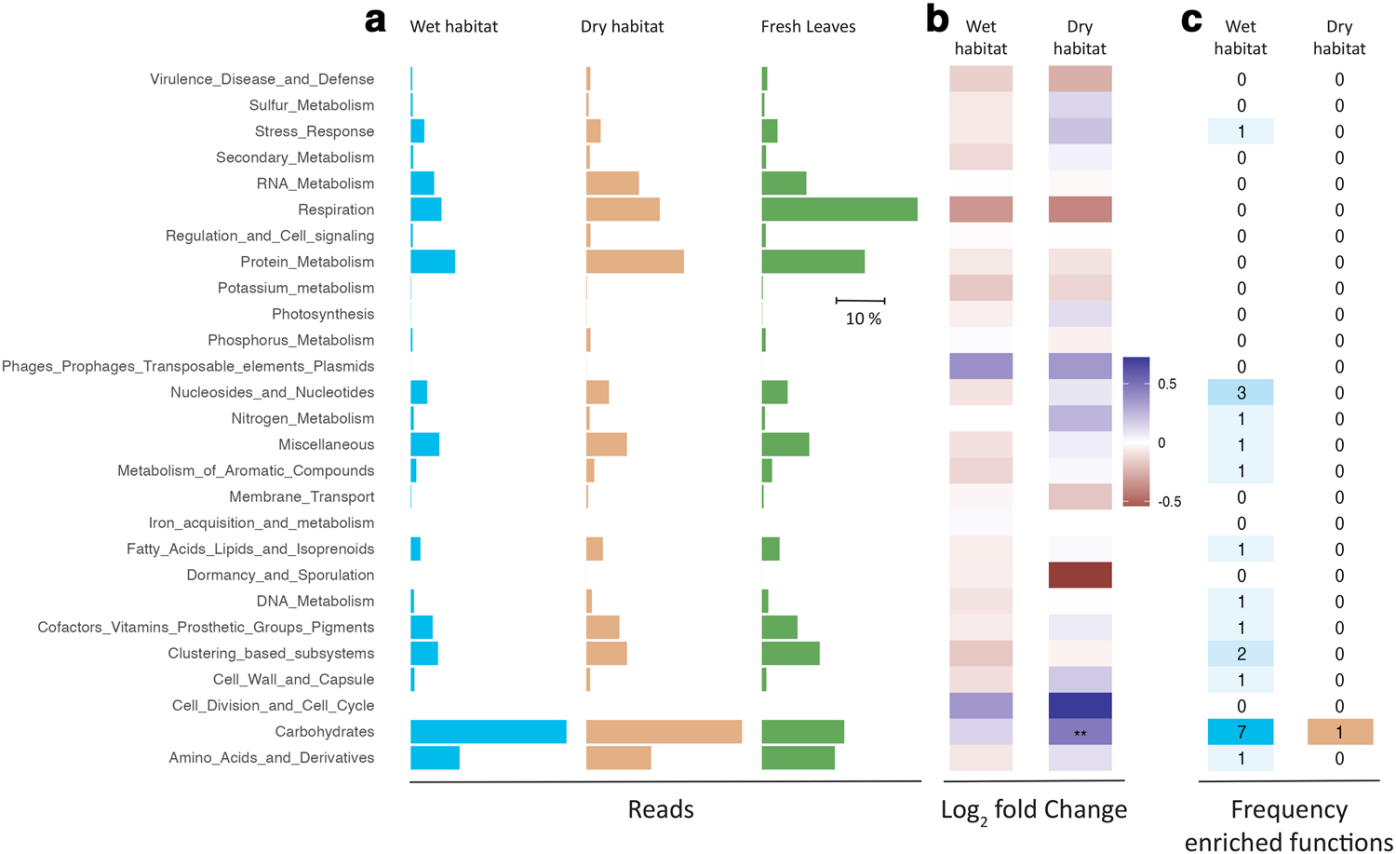
Effect of decomposition on fungal physiology at level 1 of SEED Subsystems classification: relative abundance of reads for fresh leaves or leaves decomposing in wet/dry habitats; DESeq analysis of reads from each habitat compared to fresh leaves; frequencies of enriched functions (*p* < 0.05) in wet/dry habitat.

Redundancy analysis showed no separation in fungal functional communities on fresh leaves or leaves decomposing in either dry or wet habitats (Fig. 2b). The highest number of reads in fresh leaves was observed for the functional category of Respiration. In contrast, in decomposing leaves, it was observed for Carbohydrate metabolism (Fig. 4a). Carbohydrate metabolism was the only level 1 Subsystems annotations that showed a high relative abundance of reads and a significant log2 fold change (Fig. 4b) and a difference between leaves decomposing in dry vs wet habitat (log2fold change = −1.2, *p* < 0.1).

Leaves decomposing in the wet habitat had a higher number of enriched functions, with the highest numbers observed in Carbohydrate metabolism, which was true for both habitats (Fig. 4c). SIMPER analysis revealed that Carbohydrate metabolism contributed 42% and 31% of the dissimilarity between fresh leaves and leaves decomposing in dry and wet habitat respectively. The dissimilarity between leaves decomposing in wet vs dry habitat was due to Carbohydrates (42%), Respiration (15%), Amino acids and derevatives (9%), and Protein metabolism (6%).

### Carbohydrate metabolism genes

As Carbohydrate metabolism was the most enriched level 1 annotation, we further analysed this group of genes. Drawing conclusions from shifts in upper-level classes of functions may be misleading, as multiple genes belonging to the same class can respond in opposite directions and, therefore, may result in no net effect for that class of functions. Consequently, we assessed shifts in expression at the level of individual genes to identify those unique to or significantly enriched in communities from decomposing leaves in either habitat.

**Figure 5:**
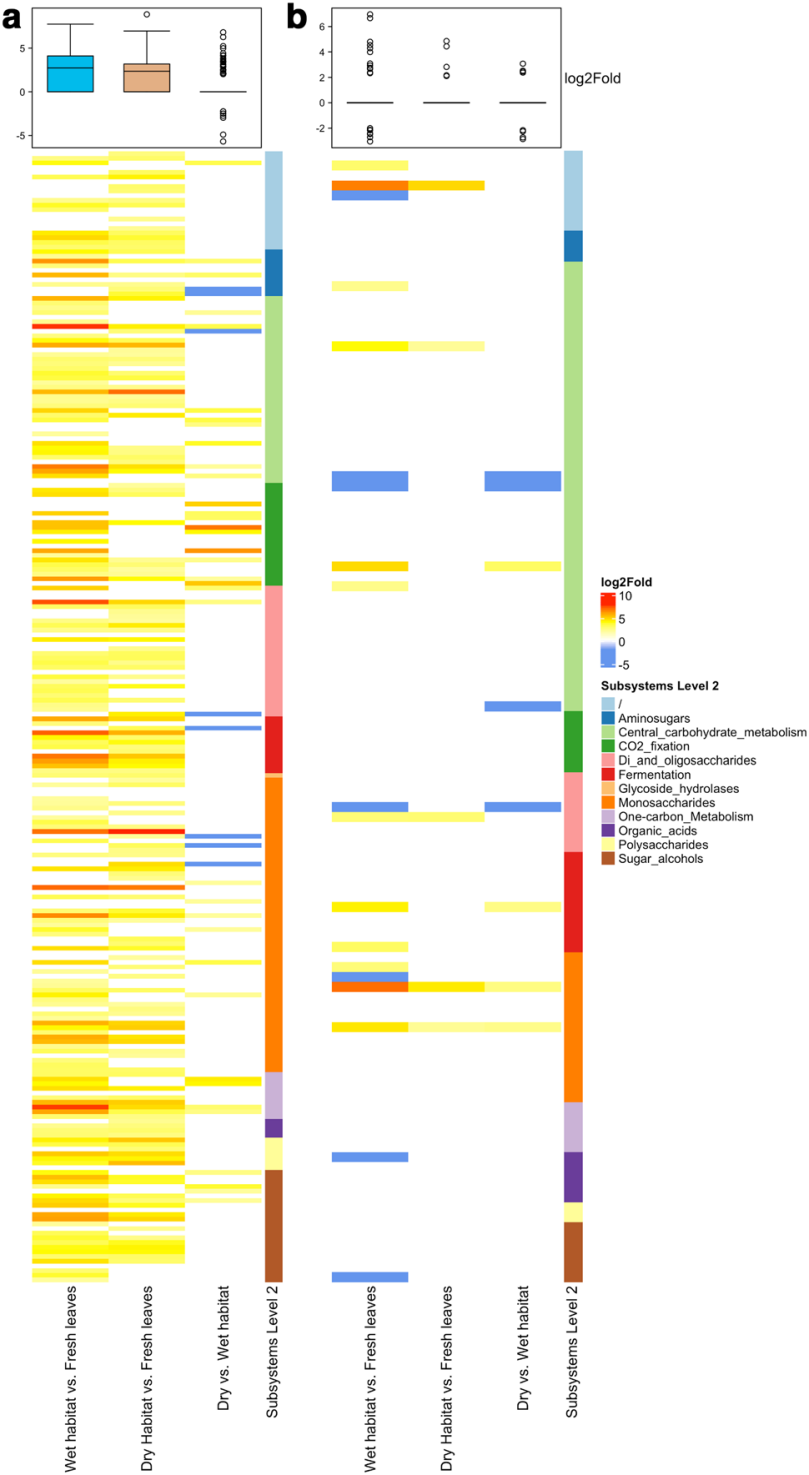
Heatmap of enriched (yellow to red) and under-enriched (blue) functions in the Carbohydrates Subsystems category for (**a**) bacterial and (**b**) fungal communities grouped according to Level 2 annotations for individual comparisons between different treatments. Functions with log2Fold change of less than two are coloured white. Boxplot represents variances of log2Fold changes for individual comparison depicted below in the heatmap column.

A comparison of functions between leaves decomposing in either habitat with fresh leaves showed that most functions were enriched (Fig. 5a).

**Figure 6:**
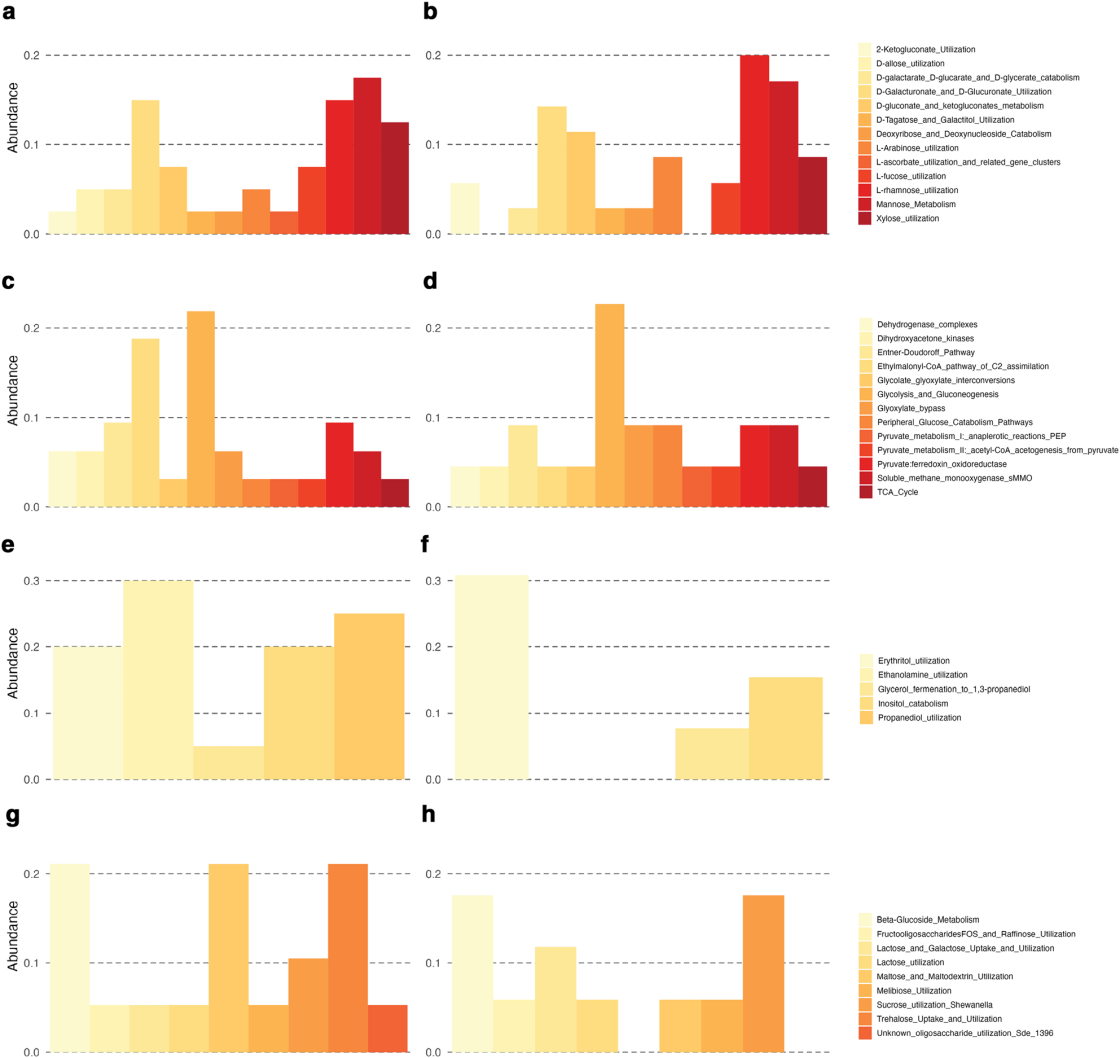
Abundance of Subsystems level 3 annotations in Carbohydrate metabolism: Monosaccharides (**a, b**), Central carbohydrate metabolism (**c, d**), Sugar alcohols (**e,f**), and Di- and Oligosaccharides (**g, h**) for leaves decomposing in wet habitat (**a, c, e, g**) or dry habitat (**b, d, e, h**) when compared to fresh leaves. Only annotations for functions with *p* < 0.01 and log2Fold change > 2 were used for the visualization.

Two-thirds of the enrichment in the Carbohydrates category was based on these Subsystem level 2 annotations: Monosaccharides (23.7–24.3%), Central carbohydrate metabolism (15.5–18.9%), Sugar alcohols (9.5–11.8%), and Di- and Oligosaccharides (11.2–11.5%). The Monosaccharides subcategory included sequences for Xylose utilization, Mannose metabolism and L-rhamnose utilization that comprised almost half of all sequences (Fig. 6a,b). In Central carbohydrate metabolism we observed a more evenly distributed abundance of individual level 3 annotations (Fig. 6c,d). Sugar alcohol metabolism contained only 4–5 level 3 categories with different abundances for leaves decomposing in dry or wet habitat (Fig. 6e,f). Subcategory Di- and Oligosaccharides included three categories with high abundances: Beta-Glucoside metabolism, Trehalose Uptake and Utilisation, and Maltose and Maltodextrin Utilization, which again comprised more than half of all sequences (Fig. 6g,h).

A comparison of individual Subsystems functions in the Carbohydrates category showed less difference in leaves decomposing in different habitats than when decomposing leaves were compared with fresh leaves. Some of the functions were under-enriched in the wet habitat when compared to the dry habitat. These are associated with categories Aminosugars (Chitin and N-acetylglucosamine utilization), Central carbohydrate metabolism (Pyruvate Alanine Serine Interconversions), Di- and oligosaccharides (Trehalose Uptake and Utilization), Fermentation (Acetoin butanediol metabolism), and Monosaccharides (2-Ketogluconate Utilization, Mannose Metabolism and Hexose Phosphate Uptake System) (Suppl. Table S2). In contrast, far more functions were enriched in the wet habitat: Aminosugars (2 functions), Central carbohydrate metabolism (eight functions), CO_2_ fixation (nine functions), Di- and oligosaccharides (two functions), Monosaccharides (six functions), One-carbon metabolism, and Sugar alcohols (four functions each) (Suppl. Table S2).

Compared to bacterial functional communities, fewer functions in the SEED Carbohydrate category were enriched in fungal functional communities when comparing decomposing leaves with fresh leaves. Furthermore, many functions showed under-enrichment, especially for the wet habitat (Fig. 5b).

**Figure 7:**
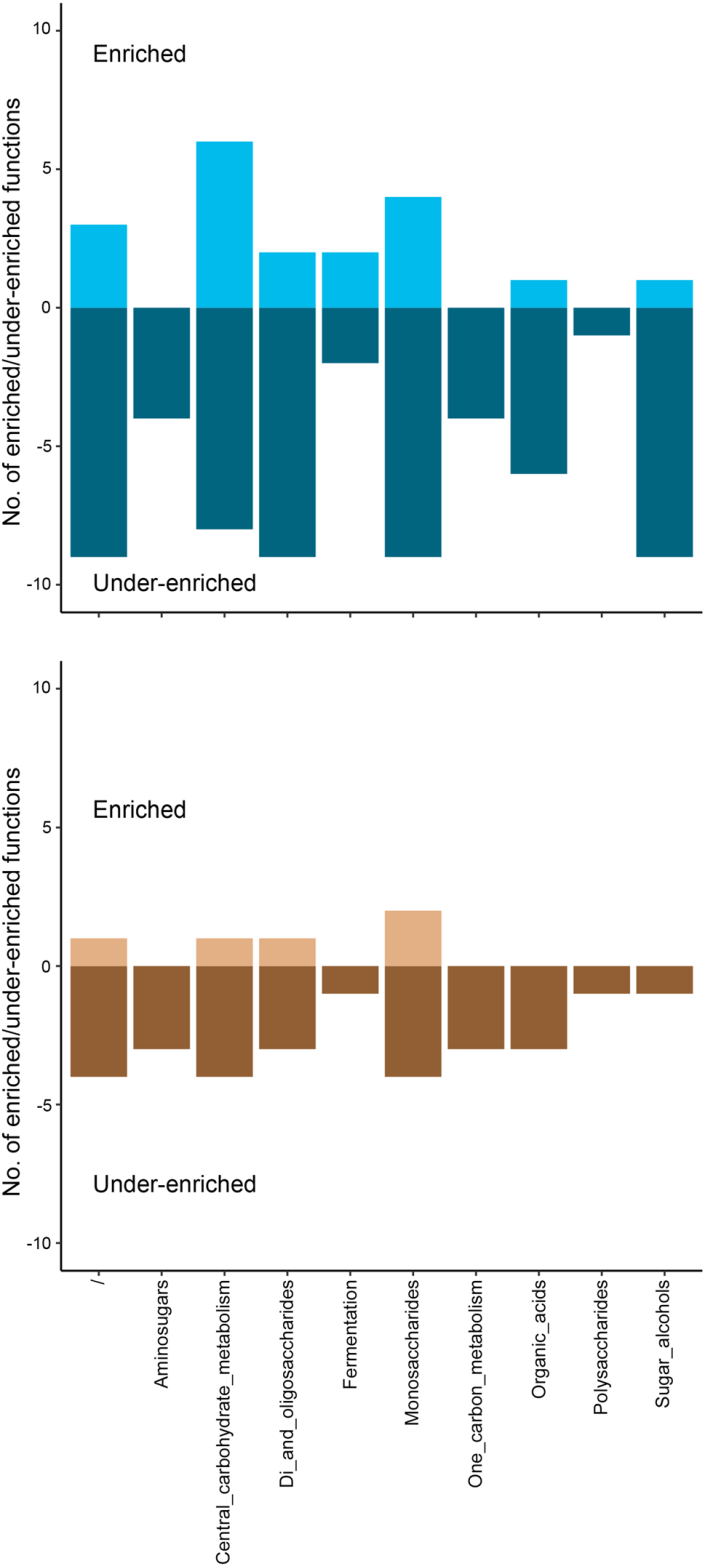
The number of enriched and under-enriched functions in fugal communities for comparison of leaves decomposing in (**a**) wet or (**b**) dry habitat vs fresh leaves. Only annotations for functions with *p* < 0.01 and log2Fold change > 2 were used for the visualization.

Enrichment of functions in the Carbohydrates category for fungal functional communities followed a similar pattern to the bacterial communities (Fig. 7). The highest number of enriched functions in the fungal communities was observed for the category Central carbohydrate metabolism in the wet habitat and Monosaccharides in the dry habitat. Far more functions were under-enriched in the wet habitat than in the dry habitat when compared to fresh leaves, with the highest numbers of under-enriched function in the categories Di- and Oligosaccharides, Monosaccharides and Unknown. In addition, in the wet habitat category, Sugar alcohols also displayed a high number of under-enriched functions. Comparison between the habitats showed only four functions that were enriched and four functions that were under-enriched in the wet habitat when compared to the dry habitat (Suppl. Table S3).

## Discussion

### Metabolic changes in microbial communities during transition to decomposition

We found distinctive functional signatures of microbial communities growing on the decomposing *P. australis* leaves. Bacterial and fungal communities showed an increase in functional α–diversity during the transition from fresh leaves to plant litter with the exception of fungal communities in the wet habitat. As plants gradually undergo senescence, the variability in phyllosphere microbes tends to incrementally rise^30^, with changes in microbial communities on leaves undergoing decomposition^31,32^ increasingly influenced by the leaves' physicochemical properties and competition between the microbes. Nevertheless, the similarity of functional α–diversity of fungal communities observed in our study between fresh leaves and leaves decomposing in the wet habitat could be specific for fungal communities on submerged plant litter, as Koivusaari et al.^33^ observed that fresh leaves and submerged litter shared 65% of fungal taxa. Although many biological functions seem redundantly distributed within decomposer communities^34^, fungal decomposer communities on submerged litter follow a unique pattern. Most taxa of Basidiomycota are absent in aquatic environments^35^, and aquatic hypomycetes that decompose the plant material in freshwater environments are suggested to have a terrestrial plant-associated life cycle phase^36,37^ and could be transfered from the leaves to the litter. As such, these specifics could reduce the rise of functional α–diversity in leaves decomposing in the wet habitat, as observed in our study.

Clustering-based subsystems and Carbohydrate metabolism had the largest quantity of annotated reads. Our observations are similar to the observations of^38^ in natural grassland soils or^39^ on maize roots, which indicates that this is probably a pattern characteristic of many biological systems and/or metagenomic studies. Many of the functions enriched in decomposing leaves were linked to carbohydrate metabolism, especially mono-, di- and oligosaccharides such as L-rhamnose, trehalose, maltose and maltodextrin, D-galacturonate, etc. These can represent the housekeeping functions, although increased expression of genes linked to the metabolism of sugars like trehalose could suggest an adaptive mechanism of compatible solute accumulation to maintain cellular osmotic balance^40^. Enrichment of genes connected with trehalose metabolism in the dry habitat further supports this.

Nevertheless, the enrichment of several of these functions is probably connected to the decomposition of *P. australis* litter. Enrichment of L-rhamnose and D-galacturonate utilization functions can be related to the degradation of rhamnogalacturonans that comprise pectin molecules of land plants^41^. Similarly, xylose is the most abundant pentose sugar (up to 25%) in lignocellulosic biomass^42^, and its efficient conversion by microbe is a major step in plant litter decomposition. Structural carbohydrates can persist in plant litter even after the first year of decay due to their close association with lignin and thus provide important substrates for the microbe communities in the litter.

In fungal functional communities, we observed a shift from a high number of Respiration connected reads to a low number of reads and an opposite shift for Carbohydrate metabolism when comparing decomposing and fresh leaves. This change in functions could be associated with changes in the fungal environment that happen during the transition from fresh to senescent and decomposing leaves. The phyllosphere is considered a low-nutrient environment, with carbon-containing nutrients acting as a major determinant of epiphytic colonization^43^. A low number of Carbohydrates annotated genes would confirm a restriction on investments into metabolic pathways that are not feasible in such a carbon-starved environment. Furthermore, the shift from fresh to decomposing leaves also has profound changes in the fungal taxonomic communities^13^. These changes in the composition of the fungal community could mean that more decomposers with higher expression of hydrolytic enzymes pointed against lignocellulosic biomass colonize plant material and explain the increase in carbohydrate metabolism.

Many functional indicators for Carbohydrates and metabolism connected to Proteins, and Amino Acids and derivatives show increased investment in substrate degradation, uptake and assimilation in microbial communities. Microbial communities in the litter may have increased investment in these resource acquisition traits to degrade the more chemically complex and diverse substrates.

In addition, the Clustering-based Subsystems category showed a high number of reads in bacterial functional communities. Clustering-based subsystem is a subsystem in which there is evidence for functional connection among the proteins, but their exact roles in the metabolic pathways are yet unknown. The high number of reads for this category in the metagenomes reveals the lack of knowledge about the microbial proteome that still exists and thus warrants further screening and orientation towards proteomic analyses.

### Community functions unique to the habitat of decomposition

Bacterial functional communities showed a high degree of separation, indicating profound differences based on the habitat where common reed leaves decompose. Fungal functional communities, in contrast, showed far fewer differences between the habitats. This lack of differences could be a result of fungal and bacterial communities responding differently to the environmental factors and their interactions^44,45^ or a larger impact of the surrounding vegetation that could serve as a source for dispersal and thus provide a unification effect between different treatments. Redondo et al.^46^observed that vegetation may be able to maintain similar fungal communities across distances and is likely the driving factor of fungal spore deposition at the landscape level.

Carbohydrates were part of the Subsystems category that showed the highest contribution to the differences between fresh leaves and leaves decomposing in dry or wet habitat and dissimilarity between leaves decomposing in both habitats and were selected for further analysis. A comparison of individual Subsystems functions in Carbohydrates showed less difference in leaves decomposing in different habitats than when decomposing leaves were compared with fresh leaves. Nevertheless, several differences in enrichments of functional traits were observed between litter decomposing in wet and dry habitats. Berlemont et al.^45^ observed that reduced precipitation decreased bacterial abundance and cellulolytic potential. As microbes convert polysaccharides into vital metabolites, such as acetate, glycerol, pyruvate, succinate, and esters, a decrease in cellulose degradation would affect several metabolic pathways. Furthermore, several differences in enrichments of functions in Central carbohydrate metabolism and Monosaccharides could be linked to different degrees of litter fragmentation in wet and dry habitats^47^ and thus changed the availability of primary substrates in the litter in both habitats. As starch and cellulose-degrading microbes lyse the initial polysaccharides, they release oligosaccharides that are accessible for many other lineages^48^. Therefore changes in the availability of the initial substrate could be felt through different metabolic pathways as changes in enrichment of functional traits. In fungal functional communities, we observed a similar pattern of under-enriched functions in leaves decomposing in wet and dry habitats. Nevertheless, the number of under-enriched functions was higher under wet conditions. Berlemont et al.^45^ also observed that fungal and bacterial communities reacted differently to seasonal patterns, with fungi preferring the dry season, which could lead to the observed under-enrichment of different functions in the wet habitat in our study.

In addition to Carbohydrates, Respiration was an additional functional trait that contributed to dissimilarity in the microbial functional communities in both habitats. Environmental moisture can significantly impact the decomposition of organic matter by affecting the diffusion of oxygen and the availability of substrates for microorganisms^49^, which is especially true for fully submerged systems. In bacterial functional communities, Nitrogen metabolism and Metabolism of aromatic compounds functions were enriched in the wet habitat when compared to the dry habitat. In submerged environments, such as aquatic ecosystems or waterlogged soils, nitrogen may become more available for plants and microorganisms due to reduced oxygen levels. Furthermore, polyphenols that are one of the main inhibitors of microbial decomposition, were found to be less effective in binding nitrogen in submerged conditions due to their solubility and leaching from the litter^50,51^. In addition, polyphenols can be degraded as part of metabolic pathways by some anaerobic microbes^52^, which would explain the enrichment of functional traits connected to metabolism of aromatic compound in the wet habitat.

Similarly, to Respiration Protein metabolism, and Amino acids and Derivatives functions were also under-enriched in the wet habitat in the fungal community, which would suggest that drier conditions were favourable for the fungal community, as already reported by Berlemont et al.^45^. Nevertheless, similarly to Grašič et al.^53^, we also observed higher decomposition rates of common reed litter under wet conditions. As such it seems that more frequent or prolonged flooding does not affect the decomposition process even if there were several negative changes in the functional traits in the fungal community. On the contrary, more frequent flooding increased the decomposition rate. This contradiction could be due to a fast recovery of fungal communities in the intermittent dry periods and/or higher decomposition by bacteria under flooded condition. In addition, we must emphasise that only 4% of all the fungal sequences were annotated with functional traits. This means that many fungal functional traits are probably still not identified and that improved knowledge of them could shift our understanding of fungal communities' behaviour under the studied conditions.

## Conclusions

To conclude, shifts in the environmental conditions due to changing precipitation patterns will alter functional microbial communities. Our results indicate that microbial communities under more frequent water stress due to fluctuating conditions could bear an additional metabolic cost. In addition, prolonged submergence seemed to have a negative impact on several functional traits in the fungal community, although the decomposition rate was not affected.

### Supplementary Information

Supplementary Information.

## Data Availability

Raw data used to plot Figs. 1, 2, 3, 4, 5, 6 and 7 have been placed in the Medeley Data repository at https://doi.org/10.17632/xh2mkrg6cv.1. The metagenomic data are freely accessible on the MG-Rast server at: https://www.mg-rast.org/linkin.cgi?project=mgp97071
