# Supplementary material for: Metagenomics reveals effects of fluctuating water conditions on functional pathways in plant litter microbial community
Source: Sci Rep. 2023 Dec 8;13:21741. doi: 10.1038/s41598-023-49044-x (PMC10709317; doi:10.1038/s41598-023-49044-x)
Supplement: Supplementary file 1 — Supplementary Information. [file 41598_2023_49044_MOESM1_ESM.docx]

Supplementary Material

## Metagenomics reveals effects of fluctuating water conditions on functional pathways in plant litter microbial community

## Matevž Likar^1,^*, Mateja Grašič^1^, Blaž Stres^2,3,4^, Marjana Regvar^1^ and Alenka Gaberščik^1^

^1^University of Ljubljana, Biotechnical Faculty, Department of Biology, Ljubljana, Slovenia

^2^ University of Ljubljana, Institute of Sanitary Engineering, Faculty of Civil and Geodetic Engineering, Ljubljana, Slovenia

^3^Jožef Stefan Institute, Biocybernetics and Robotics, Department of Automation, Ljubljana, Slovenia

^4^ National Institute of Chemistry, Department of Catalysis and Chemical Reaction Engineering, Ljubljana, Slovenia

***Correspondence:**

[matevz.likar@bf.uni-lj.si](mailto:matevz.likar@bf.uni-lj.si); Večna pot 111, 1000 Ljubljana, Slovenia, phone: +386-1-320 3 418

# Supplementary tables

**Supplementary Table S1**. Enriched functions (SEED Subsystems level 1) in *Phragmites australis* litter decomposing in dry habitat compared to the wet habitat for bacterial communities.

| Level 1 | baseMean | log2FoldChange | lfcSE | stat | pvalue | padj |
| --- | --- | --- | --- | --- | --- | --- |
| DNA_Metabolism | 150.212 | 0,0751642 | 0,022217 | 3,38 | 7,17E-04 | 2,01E-03 |
| Dormancy_and_Sporulation | 4.395 | 0,2207783 | 0,0453576 | 4,87 | 1,10E-06 | 4,50E-06 |
| Iron_acquisition_and_metabolism | 50.633 | 0,3063948 | 0,0515059 | 5,95 | 2,70E-09 | 2,19E-08 |
| Metabolism_of_Aromatic_Compounds | 53.456 | -0,2706937 | 0,0456893 | -5,92 | 3,13E-09 | 2,19E-08 |
| Nitrogen_Metabolism | 40.855 | -0,340994 | 0,0390567 | -8,73 | 2,52E-18 | 7,08E-17 |
| Phages,_Prophages,_Transposable_elements,_Plasmids | 34.573 | -0,1133913 | 0,0344439 | -3,29 | 9,95E-04 | 2,53E-03 |
| Phosphorus_Metabolism | 43.050 | -0,058358 | 0,0152314 | -3,83 | 1,27E-04 | 4,46E-04 |
| Photosynthesis | 4.127 | -0,3345661 | 0,0984943 | -3,40 | 6,82E-04 | 2,01E-03 |
| Potassium_metabolism | 31.983 | 0,0821335 | 0,0162842 | 5,04 | 5,00E-07 | 2,10E-06 |
| Respiration | 121.348 | -0,1330878 | 0,0216891 | -6,14 | 8,45E-10 | 1,18E-08 |
| Secondary_Metabolism | 9.097 | -0,1997933 | 0,0383352 | -5,21 | 2,00E-07 | 1,00E-06 |

**Supplementary Table S2**. Enriched functions (SEED Subsystems) in *Phragmites australis* litter decomposing in dry habitat compared to the wet habitat.

| Level 2 | Level 3 | Function | log2Fold |
| --- | --- | --- | --- |
| / | Sugar utilization in Thermotogales | Alpha-1,4-digalacturonate ABC transporter substrate-binding protein | 3,38 |
| Aminosugars | GlcNAc2 Catabolic Operon | Chitobiose phosphorylase EC 2.4.1. | 2,87 |
| Aminosugars | N-Acetyl-Galactosamine and Galactosamine Utilization | GALNS arylsulfatase regulator Fe-S oxidoreductase | 3,03 |
| Central carbohydrate metabolism | Ethylmalonyl-CoA pathway of C2 assimilation | Acetyl-CoA acetyltransferase EC 2.3.1.9 of ethylmalonyl-CoA pathway | 2,14 |
| Central carbohydrate metabolism | Ethylmalonyl-CoA pathway of C2 assimilation | Acyltransferase family protein associated with ethylmalonyl-CoA pathway | 3,40 |
| Central carbohydrate metabolism | Ethylmalonyl-CoA pathway of C2 assimilation | NnrU family protein in cluster with Mesaconyl-CoA hydratase | 3,52 |
| Central carbohydrate metabolism | Entner-Doudoroff Pathway | OpcA an allosteric effector of glucose-6-phosphate dehydrogenase cyanobacterial | 3,50 |
| Central carbohydrate metabolism | Particulate methane monooxygenase pMMO | Particulate methane monooxygenase B-subunit EC 1.14.13.25 | 2,51 |
| Central carbohydrate metabolism | Ethylmalonyl-CoA pathway of C2 assimilation | Putative cyclic di-GMP phosphodiesterase EAL domain protein | 3,83 |
| Central carbohydrate metabolism | Pyruvate:ferredoxin oxidoreductase | Pyruvate:ferredoxin oxidoreductase alpha subunit EC 1.2.7.1 | 2,06 |
| Central carbohydrate metabolism | Pyruvate:ferredoxin oxidoreductase | Pyruvate:ferredoxin oxidoreductase gamma subunit EC 1.2.7.1 | 2,53 |
| CO2 fixation | CO2 uptake carboxysome | Carbon dioxide concentrating mechanism protein CcmO | 4,98 |
| CO2 fixation | CO2 uptake carboxysome | Carboxysome protein CcmM | 3,16 |
| CO2 fixation | CO2 uptake carboxysome | Carboxysome protein CcmN | 3,10 |
| CO2 fixation | CO2 uptake carboxysome | Low-affinity CO2 hydration protein CphX | 6,81 |
| CO2 fixation | CO2 uptake carboxysome | NADH dehydrogenase subunit 4 Involved in CO2 fixation | 4,13 |
| CO2 fixation | Calvin-Benson cycle | Ribulose bisphosphate carboxylase EC 4.1.1.39 | 6,26 |
| CO2 fixation | CO2 uptake carboxysome | Rubisco activation protein CbbO | 2,32 |
| CO2 fixation | CO2 uptake carboxysome | putative sodium-dependent bicarbonate transporter | 2,03 |
| CO2 fixation | CO2 uptake carboxysome | ribulose 1,5-bisphosphate carboxylase/oxygenase activase | 5,22 |
| Di and oligosaccharides | Unknown oligosaccharide utilization Sde 1396 | 2nd GPH family transporter in unknown oligosaccharide utilization Sde 1396 | 2,91 |
| Di and oligosaccharides | Beta-Glucoside Metabolism | Cellobiose phosphorylase EC 2.4.1. | 2,53 |
| Monosaccharides | L-fucose utilization | L-fucose operon activator | 2,05 |
| Monosaccharides | D-SorbitolD-Glucitol and L-Sorbose Utilization | L-sorbose 1-phosphate reductase EC 1.1.1. | 2,07 |
| Monosaccharides | L-rhamnose utilization | Lactaldehyde reductase EC 1.1.1.77 | 2,03 |
| Monosaccharides | Mannose Metabolism | Mannoside ABC transport system permease protein 1 | 2,03 |
| Monosaccharides | L-rhamnose utilization | Pedicted L-rhamnose permease NCS1 Family | 3,46 |
| Monosaccharides | D-Galacturonate and D-Glucuronate Utilization | Predicted D-glucuronide-specific TRAP transporter large transmembrane component | 2,16 |
| One-carbon Metabolism | Methanogenesis | CoB-CoM heterodisulfide reductase subunit B EC 1.8.98.1 | 4,40 |
| One-carbon Metabolism | Methanogenesis | CoB-CoM heterodisulfide reductase subunit C EC 1.8.98.1 | 4,17 |
| One-carbon Metabolism | Methanogenesis from methylated compounds | Trimethylamine:corrinoid methyltransferase | 2,88 |
| One-carbon Metabolism | Serine-glyoxylate cycle | crotonyl-CoA reductase | 2,47 |
| Sugar alcohols | Inositol catabolism | 5-keto-2-deoxy-D-gluconate-6 phosphate aldolase EC 4.1.2.29 | 2,50 |
| Sugar alcohols | Propanediol utilization | CobIIalamin reductase | 3,72 |
| Sugar alcohols | Propanediol utilization | CobIIIalamin reductase | 2,05 |
| Sugar alcohols | Ethanolamine utilization | Ethanolamine utilization polyhedral-body-like protein EutM | 2,09 |

**Supplementary Table S3**. Under-enriched functions (SEED Subsystems) in *Phragmites australis* litter decomposing in dry habitat compared to the wet habitat.

| **Level 2** | **Level 3** | **Function** | **log2Fold** |
| --- | --- | --- | --- |
| Aminosugars | Chitin and N-acetylglucosamine utilization | Predicted transcriptional regulator of N-Acetylglucosamine utilization LacI family | -2,49 |
| Aminosugars | Chitin and N-acetylglucosamine utilization | predicted N-acetylglucosamine kinase glucokinase-like EC 2.7.1.59 | -4,89 |
| Central carbohydrate metabolism | Pyruvate Alanine Serine Interconversions | D-serine/D-alanine/glycine transporter | -2,49 |
| Di and oligosaccharides | Trehalose Uptake and Utilization | Trehalose operon transcriptional repressor | -2,24 |
| Fermentation | Acetoin butanediol metabolism | Acetolactate synthase small subunit EC 2.2.1.6 Xanthomonadales type | -5,67 |
| Monosaccharides | 2-Ketogluconate Utilization | Epimerase KguE | -2,23 |
| Monosaccharides | Mannose Metabolism | Fructokinase in mannoside utilization gene cluster EC 2.7.1.4 | -2,74 |
| Monosaccharides | Hexose Phosphate Uptake System | Hexose phosphate uptake regulatory protein UhpC | -2,96 |
